# Effects of COVID-19 vaccination on human fertility: a post-pandemic literature review

**DOI:** 10.1080/07853890.2023.2261964

**Published:** 2023-09-27

**Authors:** Chao Wang, Min Wang, Guanjian Li, Bing Song, Qiong Xing, Yunxia Cao

**Affiliations:** a Department of Obstetrics and Gynecology, the First Affiliated Hospital of Anhui Medical University, Hefei, China; b Department of General Office, the First Affiliated Hospital of Anhui Medical University, Hefei, China; c NHC Key Laboratory of Study on Abnormal Gametes and Reproductive Tract, Anhui Medical University, Hefei, China

**Keywords:** COVID-19, SARS-CoV-2, vaccine, fertility

## Abstract

Although vaccination with the Coronavirus disease 2019 vaccine is important and effective in the prevention of SARS-CoV-2 infection, the public expressed concerns regarding the adverse effects of vaccine on fertility. Some reviews have focused on it, they have been unable to collect sufficient research data because of the earlier publication period. As relevant evidence has gradually increased, we reviewed these studies from the perspectives of males, females with or without pregnancy, and different vaccine types. The results suggest that although males may experience fluctuations in semen parameters within their physiological ranges after receiving the vaccine, it has not yet reached a level of influence on the partner’s pregnancy probability. As to female without pregnancy, it is believed that vaccination will not affect fertility; however, more research is needed to explore the short-term impact. Vaccination during any trimester is considered safe in pregnant women.

## 1. Introduction

In 2019, severe respiratory diseases emerged and rapidly spread worldwide. Coronavirus disease 2019 (COVID-19), caused by the severe acute respiratory syndrome coronavirus 2 (SARS-CoV-2), is particularly burdensome. It has been estimated that 18.2 million people died worldwide between January 1, 2020, and December 31, 2021, because of the COVID-19 pandemic (as measured using excess mortality rates) (Collaborators 2022). Countries have taken numerous measures to control the pandemic, among which the COVID-19 vaccination has been important and effective [62, 78]. To date, 69.9% of the world’s population have been vaccinated with at least one dose of COVID-19 vaccine, and 13.38 billion doses of vaccine have been administered (https://ourworldindata.org/covid-vaccinations. Accessed 10 April, 2023). As the number of vaccinated individuals increase, concerns regarding vaccine safety continue to increase. As the population is generally vulnerable to COVID-19, almost everyone is an adaptation of the vaccine, regardless of sex, age, race, or occupation, which naturally includes individuals who have a pregnancy plan in the short term. The reason for their concern is that the COVID-19 vaccine development cycle is short, and more importantly, the related clinical trials did not include pregnant women or those who may have pregnancies in the short-term. Concerns about the adverse effects on fertility have become the main cause of vaccination hesitation in the United States [21]. Claims that COVID-19 vaccine cause infertility and miscarriage in pregnant women have been widely shared on social media [59, 71]. These erroneous messages cause infertile patients to hesitate to receive a vaccine [1]. A research report has shown that the vaccination rate of women receiving assisted reproductive technology (ART) treatment is significantly lower than that of the general population [85]. Fortunately, some researchers have discussed the impact of COVID-19 vaccine on human fertility [16, 90, 32, 65, 54, 35]. However, owing to the earlier publication period, they were unable to summarize sufficient research reports. With the increasing administration of vaccines, the relevant evidence is also gradually increasing. Therefore, we reviewed these articles and analyzed the impact of the COVID-19 vaccine on human fertility from the perspective of males, females with or without pregnancy, and different vaccine types. The results of this review will improve our understanding of the effects of the COVID-19 vaccination on fertility.

## 2. Design principles of different types of COVID-19 vaccine

It took a very short time from the design to clinical application of COVID-19 vaccine, which is an unprecedented speed in the history of vaccine. The COVID-19 vaccine currently being used can be roughly divided into the following categories: messenger RNA (mRNA) vaccine, inactivated vaccine, viral vector vaccine and other rare types. Messenger RNA (mRNA) vaccine: the mRNA sequence corresponding to the SARS-CoV-2 spike glycoprotein (S protein) was synthesized by gene synthesis technology, and the synthesized mRNA sequence was packaged with nanoparticles and other carriers to protect the integrity of the sequence, and then injected into the human body. Human cells read the mRNA sequence and produce pathogen antigen protein to induce immune response. Inactivated vaccine: the principle of inactivated virus preparation is to carry out viral culture and amplification *in vitro*, inactivate the virus by heating or chemical methods, retain antigen components to induce immune response of the body, and add adjuvant such as aluminium hydroxide to improve immunogenicity. Viral vector vaccine: the SARS-CoV-2 S protein gene is recombined into a modified non-pathogenic live virus vector, usually an adenovirus vector. The genetically recombinant adenovirus expresses the SARS-CoV-2 S protein antigen *in vivo*, inducing the body to produce an immune response. Other types, such as the recombinant subunit vaccine used in China: The gene of receptor binding domain (RBD) of the S protein is recombined into the gene of Chinese hamster ovary (CHO) cells so they can express it and form RBD dimer *in vitro*. As well as, aluminium hydroxide adjuvant is added to improve immunogenicity and induce immune response of the body.

## 3. Effects of COVID-19 vaccination on male fertility

Angiotensin-converting enzyme 2 (ACE2) mediates the entry of SARS-CoV-2 into host cells. It is highly expressed in the testes, making it a target organ for SARS-CoV-2 infection. One study found that SARS CoV-2 infection can lead to the enhancement of the immune response in the testis and the occurrence of autoimmune orchitis [47]. This may explain the decrease in sperm parameters observed in many infected patients [22, 45, 87]. Therefore, researchers were curious about whether a similar phenomenon would occur after COVID-19 vaccination. Table 1 presented the effects of mRNA vaccines on human semen parameters in different studies.

**Table 1.** Effects of different types of COVID-19 vaccine on male semen.

	References	Study design	Vaccine type	Sample size	History of SARS CoV-2 infection	Semen testing time after vaccination	Control	Main outcomes
1	[49]	Prospective cohort study	mRNA vaccine	75 fertile men	No	Average 37 days (1–2 months) after the second dose	The WHO reference ranges	No significant differences
2	[69]	Retrospective cohort study	mRNA vaccine	72 IVF patients	No	Average 71 days (40.5-104.8 days) after the first dose	Self-control	No significant differences
3	[64]	Prospective cohort study	mRNA vaccine	47 hospital staffs	No	3 months after the first dose	Self-control	No significant differences
4	[43]	Retrospective cohort study	mRNA vaccine	58 IVF patients	No	6–14 months after the first dose	Self-control	No significant differences
5	[8]	Prospective cohort study	mRNA vaccine	33 donors at the sperm bank	Unknown	At least 72 days after the second dose	Self-control	Total sperm count and total motile count increased after the vaccination. Percent of motile sperm did not change.
6	[31]	Prospective cohort study	mRNA vaccine	45 healthy volunteers	No	A median of 75 days after the second dose	Self-control	The median sperm concentration, total motile sperm count, semen volume and sperm motility significantly increased
7	[2]	Prospective cohort study	mRNA vaccine	60 IVF patients	Unknown	At least 90 days after the second dose	Self-control	No differences in semen volume, sperm concentration, and morphology. Progressive motility significantly increased.
8	[20]	Prospective cohort study	mRNA vaccine	12 healthy volunteers	No	3 months (T1), at least 9 months (T2) after the second dose	Self-control	No significant differences
9	[30]	Retrospective cohort study	mRNA vaccine	37 donors at the sperm bank	No	15–45 days (T1), 75–125 days (T2) and at least 145 days (T3) after second dose	Self-control	Temporary sperm concentration and total motile count deterioration 3 months after vaccination followed by later recovery
10	[24]	Retrospective cohort study	Inactivated vaccine	519 fertile men	No	≤ 90 days (T1), >90 days (T2) after the first dose	Unvaccinated group	Most of the semen parameters remained unchanged; T1 group have decreased total sperm motility
11	[86]	Retrospective cohort study	Inactivated vaccine	260 IVF patients	No	Average 80.6 days after the first dose	Unvaccinated group	No significant differences
12	[91]	Retrospective cohort study	Inactivated vaccine	43 donors at the sperm bank	No	Within 21 days(T1), 60 days (T2) after the first dose	Self-control	No significant differences
13	[83]	Prospective cohort study	Inactivated vaccine	1219 IVF patients	No	A median of 71 days after the second dose	Self-control	No significant differences
14	[88]	Retrospective cohort study	Inactivated vaccine	128 men	Unknown	Average 87.5 days after the second dose	Self-control	No significant differences
15	[56]	Prospective cohort study	76% mRNA vaccines, 20% viral vector vaccine, 2% a mixed vaccine, 2% unknown	101 men	Unknown	2.3 ± 1.5 months after the second dose	Self-control	Semen volume significantly decreased. Sperm concentration, progressive motility, and total motile sperm count significantly increased
16	[6]	Prospective cohort study	44% mRNA vaccine, 32% viral vector vaccine, 24% mixed vaccine	100 volunteers	No	1 month after the second dose	Self-control	Progressive sperm motility increased significantly
17	[68]	Retrospective cohort study	87.7% mRNA vaccine, 10.4% viral vector vaccine, 1.9% mixed vaccine	106 IVF/IUI patients	No	59 days after the second dose	Self-control	No significant differences
18	[46]	Retrospective cohort study	33.3% mRNA vaccine, 33.3% inactivated vaccine, 33.3% viral vector vaccine	70 infertile men	No	Unknown	Unvaccinated group	The viral vector vaccine caused a decrease in morphology as well as an increase in DNA fragmentation index
19	[57]	Retrospective cohort study	Viral vector vaccine	53 infertility patients	No	82 days after the second dose	Self-control	No significant changes in semen parameters, except for a moderate decline in sperm morphology

IVF, *in vitro* fertilization; IUI, Intrauterine insemination.

### 3.1. Messenger RNA (mRNA) vaccines

The COVID-19 vaccine used worldwide is mainly an mRNA vaccine; therefore, much attention has been focused on the impact of mRNA vaccines. Lifshitz et al. believed that Pfizer’s mRNA COVID-19 vaccine was safe because they analyzed the semen samples of 75 fertile males and found that the semen parameters after COVID-19 vaccination were mainly within the normal reference range specified by the World Health Organization [49]. However, the lack of control in this study affects the credibility of the conclusions. Many studies have used a self-controlled approach to analyze the changes in semen parameters before and after vaccination. The mRNA vaccine was the only vaccine included in reports by Safrai M et al. [69], Olana S et al. [64], and Karavani G et al. [43]. In their study, there were no statistically significant changes in the semen parameters after vaccination. The other three studies included only mRNA vaccines; however, their conclusions differed. They found an increase in certain semen parameters after the administration of the COVID-19 mRNA vaccine. These parameters included semen volume, total sperm count, and total motile count [8, 31, 2]. Seven of the eight patients with initial oligozoospermia showed an increase in sperm concentration into the normozoospermic range [31]. Although the above study described the time interval between vaccination completion and semen analysis, it did not distinguish between individuals at different time intervals. This may have limited the clinical significance of these findings. Diaz et al. analyzed semen samples before mRNA vaccine vaccination and samples from 3 and 9 months after vaccination and found no significant differences in any parameter between time measurements [20]. It should be noted that only 12 volunteers participated in this study, and the small sample size was a limitation. Gat et al. expanded the number of participants to 37, and each participant provided one to three semen samples for analysis at different time points and found a temporary decline in sperm concentration and motile count at 3 months post-vaccination, followed by recovery [30].

### 3.2. Inactivated, viral vector, and other types of vaccines

Relatively little research on inactivated COVID-19 vaccine is available, mainly reported by scholars in China, who have the most applications for inactivated COVID-19 vaccine. Two retrospective studies included an unvaccinated population in the control group, and the results showed that the population vaccinated with inactivated COVID-19 vaccine had comparable semen parameters and pregnancy rates [24, 86]. Like Gat et al.’s report on mRNA vaccine, Dong et al. also found a deterioration in semen parameters in the short term, the population within 90 days of vaccination displayed decreased total sperm motility compared to that in the unvaccinated population [24]. We speculate that the fever after vaccination may be the reason for the parameter deterioration, since researches had proved that fever can temporarily impair sperm count and quality [72]. Furthermore, fever had been recognized as one of the most common side effects of various types of COVID-19 vaccine [41]. In the report from Dong et al. they reported various side effects after vaccination. Unfortunately, they did not focus on these vaccination populations with side effects, so it cannot be confirmed that whether this parameter change was attributed to the fever or other side effects. After using the self-control method, researchers reached the same conclusion that there was no significant change in parameters such as sperm concentration, progressive motile sperm count, and total motile sperm count after the inactivated COVID-19 vaccine was administered [91, 83; 37, 88]. In a prospective survey conducted by Massarotti et al. post-vaccination semen samples were found to have a significantly decreased volume and increased sperm concentration, progressive motility, and total motile sperm count. Seventeen patients diagnosed with oligozoospermia according to the World Health Organization reference ranges had their sperm concentration increased to the normal range after receiving the vaccine [11, 56]. A similar phenomenon was observed by Alenzi et al. who found that progressive sperm motility increased significantly after vaccination. The serum testosterone levels also increased [6]. The vaccine types included in both studies were the mRNA and viral vector COVID-19 vaccine. However, they did not conduct a subgroup analysis of the vaccine types. Reschini et al. added this point and revealed that exposure to vaccination did not alter semen parameters; even when conducting a subgroup analysis of vaccine types (mRNA or viral vector vaccines), the conclusion did not change [68]. Lestari et al. also included multiple vaccines in their study, and they conducted subgroup analysis on different vaccine types. However, this study showed the drawbacks of viral vector vaccine in causing a decrease in morphology as well as an increase in sperm DNA fragmentation index [46]. In a study by Meitei et al. males who received viral vector vaccines were the only subjects, and no significant changes in semen parameters were observed, except for a moderate decline in sperm morphology [57].

Semen parameters are key indicators, and female pregnancy rate is a direct indicator of male fertility. Although there has been little research on this direct indicator, researchers have revealed that male vaccination does not affect a partner’s pregnancy rate [86, 83, 52]. Overall, the COVID-19 vaccine did not have a significant effect on the male semen parameters. Although some studies have found statistically significant changes, these changes are characterized by parameter improvements. The changes in these data may originate from fluctuations within the normal physiological range of the semen, and the duration of abstinence before semen retrieval may explain these variations.

## 4. Effects of COVID-19 vaccination on female fertility

Irregular menstruation and vaginal bleeding are considered side effects of COVID-19 and vaccination [84, 39]. This may be due to the post-vaccination systemic inflammatory reactions that affect ovarian function. Do these reactions affect the fertility rate?

### 4.1. Messenger RNA vaccines

A small sample study showed that six women who received mRNA vaccines exhibited IgG levels similar to those of COVID-19 patients in the follicular fluid and serum. Compared to patients without COVID-19 and without vaccination, their follicles showed no differences in quality parameters, such as estrogen, progesterone, and heparin sulfate proteoglycan 2, as well as in the number and maturity of oocytes obtained [9]. In a study by Odeh Natour et al. 37 individuals who received the mRNA COVID-19 vaccine had follicular fluid and serum parameters similar to those of unvaccinated individuals [63]. Serum anti-mullerian hormone (AMH) concentration is an important indicator of ovarian reserve, and it is reported that the use of mRNA vaccines does not affect serum AMH concentration [34, 58]. To some extent, these studies have emphasized the safety of COVID-19 mRNA vaccines at the molecular level. Female fertility is usually evaluated through their performance in *in vitro* fertilization (IVF), as they cannot easily obtain gametes for research like male can. We can find some phenomena discovered by researchers in ART outcomes in Table 2. Orvieto et al. originally reported that administering mRNA vaccines did not alter ovarian stimulation parameters or embryo quality in infertile patients [66]. Additional studies have confirmed that mRNA vaccines do not affect IVF pregnancy rate [70, 14, 7, 3]. Although the outcomes of fresh and frozen embryo transfers have been discussed separately, the conclusion remains unchanged [40, 5]. Karavani et al. also considered the possible impact of time intervals after vaccination, and the results showed similar numbers of retrieved and mature oocytes in different time interval groups [42].

**Table 2.** Effects of different types of COVID-19 vaccine on ART outcomes.

	References	Study design	Vaccine type	Sample size	Male vaccination	History of SARS CoV-2 infection	ART treatment after vaccination	Control	Main outcomes
1	[66]	Retrospective cohort study	mRNA vaccine	72 cycles	Yes	Unknown	7-85 days after the second dose	Self-control	No significant differences
2	[70]	Retrospective cohort study	mRNA vaccine	84 cycles	Unknown	No	Average 131.8 days after the first dose	Unvaccinated group	No significant differences
3	[14]	Retrospective cohort study	mRNA vaccine	230 IVF cycles	21 of 29 males who reported vaccination information vaccinated	Unknown	>7 days after the second dose	Unvaccinated group	No significant differences
4	[7]	Retrospective cohort study	mRNA vaccine	400 IVF cycles	Unknown	No	14-68 days after the second dose	Unvaccinated group	No significant differences
5	[3]	Retrospective cohort study	mRNA vaccine	1205 IVF cycles	Unknown	Unknown	>14 days after the second dose	Unvaccinated group	No significant differences
6	[40]	Retrospective cohort study	mRNA vaccine	280 IVF cycles	Unknown	Unknown	Average 93 days after the second dose	Unvaccinated group	No significant differences
7	[5]	Retrospective cohort study	mRNA vaccine	936 IVF cycles	Unknown	18.4% infected in vaccinated group, none in unvaccinated group	Average 79 days after the second dose	Unvaccinated group	No significant differences
8	[42]	Retrospective cohort study	mRNA vaccine	232 IVF cycles	Unknown	No	1-3, 3–6, 6–9 and 9–13 months after the first dose	Unvaccinated group	No significant differences
9	[85]	Retrospective cohort study	Inactivated vaccine	1583 IVF cycles	80.7% vaccinated	No	≤30 days (27.5%), 31–60 days (38.4%) and ≥61 days (34.1%) after the second dose	Unvaccinated group	No significant differences
10	[15]	Retrospective cohort study	Inactivated vaccine	2091 IVF cycles	70.4% vaccinated	No	Unknown	Unvaccinated group	No significant differences
11	[52]	Retrospective cohort study	Inactivated vaccine	802 IVF cycles	48.5% vaccinated	No	Unknown	Unvaccinated group	No significant differences
12	[36]	Retrospective cohort study	Inactivated vaccine	133 PGT cycles	Unknown	No	Average 126.5 days after the second dose	Unvaccinated group	No significant differences
13	[50]	Retrospective cohort study	Inactivated vaccine	730 IVF cycles	Unknown	No	Average 72.4 days after the second dose	Unvaccinated group	No significant differences
14	[73]	Prospective cohort study	Inactivated vaccine	3052 IVF cycles	Unknown	No	≤30 days (5.2%), 31-60 days (8.7%), 61-90 days (15.7%), and ≥91 days (70.3%) after the first dose	Unvaccinated group	Receipt of the first inactivated vaccine dose 60 days or less before fertilization treatment is associated with a reduced rate of pregnancy
15	[89]	Retrospective cohort study	19.4% Viral vector vaccine, 79°2% inactivated vaccine, 1.4% recombinant subunit vaccine	2505 IVF cycles	91% vaccinated in vaccination group, none in unvaccinated group	Unknown	Unknown	Unvaccinated group	No significant differences
16	[67]	Retrospective cohort study	13.5% Viral vector vaccine, 86.5% mRNA vaccine	1700 IVF cycles	Unknown	Unknown	Approximately 2 months after the second dose	Self-control	No significant differences
17	[80]	Prospective cohort study	1.6% Viral vector vaccine, 95.7% inactivated vaccine, 2.7% recombinant subunit vaccine	4185 IUI cycles	46.9% vaccinated	Unknown	154 days after the first dose in clinical group, 146 days in nonclinical group	Unvaccinated group	No significant differences
18	[23]	Prospective cohort study	93.7% Inactivated vaccine, 6.3% recombinant subunit vaccine	554 IVF cycles	91.1% vaccinated	No	<3 months (36.2%), 3-6 months (57.5%), and >6 months (6.3%) after complete vaccination	Unvaccinated group	No significant differences
19	[88]	Retrospective cohort study	Inactivated vaccine	1000 IUI cycles	Unknown	Unknown	<3 months (27.3%), ≥3 months (72.7%) after the last dose	Unvaccinated group	No significant differences

ART, assisted reproductive technology; IVF, *in vitro* fertilization; PGT, preimplantation genetic testing; IUI, Intrauterine insemination.

### 4.2. Inactivated, viral vector, and other types of vaccines

Many retrospective studies on the impact of COVID-19 inactivated vaccine on IVF have shown that administering inactivated vaccine before ovarian stimulation did not affect the results of ovarian stimulation, embryonic development, and pregnancy rate [85, 89, 15, 52]. Huang et al. provided evidence of the safety of inactivated vaccines in terms of embryo ploidy rate [38]. In another study, individuals with different time intervals after vaccination had similar rates of oocyte retrieval, good-quality embryos, and clinical pregnancy rate [36, 50]. However, compared with longer time intervals, the ongoing pregnancy rate of vaccinated individuals within 60 days may be lower [73]. Compared with mRNA and inactivated vaccines, the population using viral vector vaccines is relatively small. Some studies included a small number of people who used viral vector vaccines, and in their subgroup analysis, it was found that viral vector vaccination did not affect the number of oocytes, high-quality embryos, or pregnancy rates of women [89, 67]. The recombinant subunit vaccine produced by a Chinese biological company is the first approved recombinant COVID-19 protein vaccine in China. Animal experiments in rats have shown that the recombinant subunit vaccine did not affect the mating performance, fertility or reproductive performance and embryonic development [75]. To date, no adverse effects of recombinant vaccines in IVF have been reported [80; 23].

Intrauterine insemination (IUI) simulates a natural conception by injecting semen into the uterus. Compared with the IVF study, the study on the impact of the COVID-19 vaccine on IUI had a greater reference value for the normal population. A retrospective study in China showed that after receiving the COVID-19 inactivated vaccine, no negative impact was found on IUI cycles [88]. Our previous national multicenter prospective study, which mainly included inactivated vaccines, also showed that the IUI pregnancy rate was not affected by the different COVID-19 vaccines or the time interval from vaccination to IUI [80]. Similar results were observed in the non-ART populations. Hillson et al. analyzed data from four clinical trials that included both mRNA and viral vector vaccines. All the volunteers had negative urine HCG test results before participating in the trial. The vaccinated and control groups reported comparable pregnancy rates during the trial period with no significant differences in miscarriage rates [33].

## 5. Effects of COVID-19 vaccination on pregnancy

Owing to changes in specific physiological conditions, including immunosuppression and high metabolic status, pregnant women are at a higher risk of COVID-19 infection. In pregnant women with severe COVID-19, the number of Th17 cells increased significantly, whereas the proportion of Tregs decreased. An imbalance in Treg/Th17 cells may lead to a systemic inflammatory response by inducing the uncontrolled release of proinflammatory cytokines [61]. Systemic inflammation increases the incidence of pregnancy complications such as pregnancy loss, gestational hypertension, and gestational diabetes [44, 19, 25]. In addition, the likelihood of intensive care unit (ICU) admission and ventilation increased in women with COVID-19 [81]. Despite these adverse effects of COVID-19, pregnant women still choose to delay or refuse vaccination because of concerns regarding vaccine safety during pregnancy [10]. Table 3 displayed the effects of different types of COVID-19 vaccine on pregnancy.

**Table 3.** Effects of different types of COVID-19 vaccine on pregnancy.

	References	Study design	Vaccine type	Sample size	History of SARS CoV-2 infection	Timing of vaccination	Control	Main outcomes
1	[74]	Retrospective cohort study	mRNA vaccine	3958 pregnant women	2.0% infected	First dose: 2.3% in Ta, 28.6% in Tb, 43.3% in Tc, 25.7% in Td	Incidences reported before the Covid-19 pandemic	No significant differences
2	[26]	Prospective cohort study	mRNA vaccine	894 pregnant women	Unknown	First dose: 3.6% in T0, 4.5% in T1, 69.7% in T2 and 22.3% in T3	Incidences reported before the Covid-19 pandemic	No significant differences
3	[13]	Prospective cohort study	mRNA vaccine	313 pregnant women	7.4% infected	First dose: 30% in Tα, 46% in Tβ, 24% in Tγ	Unvaccinated group	No significant differences
4	[28]	Retrospective cohort study	99.8% mRNA vaccine, 0.2% unknown	97590 pregnant women	3.4% infected	First dose: 0.9% in Tα, 35.5% in Tβ, 63.6% in Tγ	Unvaccinated group	No significant differences
5	[77]	Retrospective cohort study	99.3% mRNA vaccine, 0.7% viral vector vaccine	2002 pregnant women	10.6% infected	First dose: a median of 32 weeks; Completed vaccination: a median of 35 weeks	Unvaccinated group	No significant differences
6	[55]	Retrospective cohort study	98.3% mRNA vaccine, 1.7% viral vector vaccine	157521 pregnant women	10.1% infected	First dose: 3.9% in T0 + T1, 45.6% in T2, 50.4% in T3	Unvaccinated group	No significant differences
7	[79]	Retrospective cohort study	mRNA vaccine	4399 pregnant women	None infected	First dose: a mean of 7.5 weeks before delivery second dose: 5.4 weeks before delivery	Unvaccinated group	No significant differences (subgroup analysis: two doses of vaccines were associated with longer gestational weeks and higher newborn birth weight)
8	[51]	Retrospective cohort study	95.8% mRNA vaccine, 4.2% viral vector vaccine	46079 pregnant women	3.4% infected	First dose: 1.7% in Tα, 36.5% in Tβ, 61.8% in Tγ	Unvaccinated group	No significant differences
9	[28]	Retrospective cohort study	99.7% mRNA vaccine, 0.3% unknown	85162 pregnant women	3.9% infected	First dose: 12.1% in Ta + Tb, 48.1% in Tc, 39.8% in Td	Unvaccinated group	No significant differences
10	[60]	Retrospective cohort study	mRNA vaccine	15865 pregnant women	4.0% infected	Unknown	Unvaccinated group	Receipt of the mRNA vaccine was associated with a lower rate of several adverse pregnancy outcomes
11	[17]	Retrospective cohort study	mRNA vaccine	3094 pregnant women	11.0% infected	First dose: All in the first trimester	Unvaccinated group	No significant differences
12	[15]	Retrospective cohort study	Inactivated vaccine	2091 FET women	None infected	First dose: Between obtaining embryos and achieving pregnancy	Unvaccinated group	No significant differences
13	[53]	Prospective cohort study	Inactivated vaccine	967 pregnant women with natural conception	None infected before the first trimester	First dose: All in Ta + Tb	Unvaccinated group	No significant differences
14	[48]	Prospective cohort study	Inactivated vaccine	253 pregnant women	Unknown	First dose: 71% within 3 months before their LMP and 39% after LMP	Unvaccinated group	No significant differences
15	[55]	Retrospective cohort study	98.3% mRNA vaccine, 1.7% viral vector vaccine	157521 pregnant women	10.1% infected	First dose: 3.9% in T0 + T1, 45.6% in T2, 50.4% in T3	Unvaccinated group	No significant differences
16	[12]	Retrospective cohort study	90.7% mRNA vaccine, 9.3% viral vector vaccine	1328 pregnant women	1.4% infected	First dose: 14.3% in T2, 85.7% in T3	Unvaccinated group	No significant differences
17	[4]	Retrospective cohort study	88.3% mRNA vaccine, 11.7% viral vector vaccine	264927 pregnant women	Unknown	First dose: 78.8% in Ta + Tb + Tc, 21.2% in Td	mRNA vaccine group	Obstetric outcomes of pregnant women appeared to be worse with the viral vector vaccine than with the mRNA vaccine

Ta, within 30 days before last menstrual period; Tb, <14 weeks of gestation; Tc, ≥14 and <28 weeks of gestation; Td, ≥28 weeks of gestation; T0, 7 days before last menstrual period to 13 days after last menstrual period; T1, 14 days after last menstrual period and <12 weeks of gestation; T2, ≥12 and <28 weeks of gestation; T3, ≥28 weeks of gestation; Tα, from pre-conception to13 weeks of gestation; Tβ, 14–28 weeks of gestation; Tγ, 29 weeks of gestation till birth; LMP, last menstrual period; FET, frozen embryo transfer.

### 5.1. Messenger RNA vaccines

The New England Journal of Medicine (NEJM) reported preliminary findings of the mRNA Covid-19 vaccine safety in pregnant women. Pregnant women who received the mRNA vaccine during pregnancy had similar outcomes, including the rate of pregnancy loss, live births, preterm births, and small size for gestational age, compared to those before the COVID-19 pandemic [74]. In this report, some individuals were already > 20 gestational weeks at the time of the first eligible dose, which may have led to inaccurate estimates of outcomes, especially pregnancy loss rates, because pregnancy is most likely to be lost during the first trimester. However, an observational cohort study from Switzerland used a prospective survey method, which can avoid this bias, and reached a conclusion similar to that reported by the NEJM, that is, vaccinated pregnant women did not experience more adverse pregnancy or neonatal outcomes [26]. Most reports in literature support this conclusion [13, 27, 77, 55]. Owing to the early timing of these studies, not all individuals completed the two doses of vaccine injections. Wainstock et al. found that the application of two doses of vaccines was associated with longer gestational weeks and higher newborn birth weight [79]. All vaccinations were administered during the second or third trimester of pregnancy in this study; therefore, some patients completed delivery before receiving a second dose. Therefore, we believe that a longer gestational age allows sufficient time to complete the second dose of the vaccine. Other researchers did not support the results of Wainstock et al. as pregnant women with different doses (1 or 2 doses) and timing (2nd or 3rd trimester) of vaccination did not show significant differences in the incidence of premature birth and small size for gestational age at birth [51, 28]. Morgan et al. enrolled only pregnant women who had completed two doses of mRNA vaccines. Vaccines can help reduce the occurrence of adverse pregnancy outcomes, including perinatal death, premature birth, neonates with very low birth weight, and neonatal intensive care unit admissions [60]. In the aforementioned reports, vaccines were administered mainly during the second and third trimesters of pregnancy. However, the first trimester is a critical period for fetal development and is the most common period of pregnancy loss. Fortunately, mRNA COVID-19 vaccination during the first trimester did not increase the risk of pregnancy loss compared to non-vaccinated pregnant women [17]. Further research focusing on other adverse pregnancy complications in women who receive vaccines only during the first trimester is required.

### 5.2. Inactivated, viral vector, and other types of vaccines

Although the number is small, studies of other types of COVID-19 vaccine have reported their safety during pregnancy. Experiments on mice showed that inactivated vaccination during pregnancy did not undermine the maternal body weight, live birth rate, or growth and development indicators of the offspring [50]. In humans, inactivated vaccines did not affect newborn birth length and weight [15]. The risk of miscarriage, small for gestational age, gestational diabetes mellitus, preterm birth, and hypertensive disorders of pregnancy did not increase with inactivated COVID-19 vaccination [53]. Owing to the absence of differential complications during pregnancy and delivery between vaccinated and non-vaccinated pregnant women, Li et al. extended the safe application time of inactivated vaccines for pregnant women to three months before the last menstrual period and throughout the entire pregnancy period [48]. AZD1222, an adenoviral vector vaccine developed by Oxford University and AstraZeneca, has been shown to have no effect on the fertility of pregnant mice [76]. We have seen some safety reports on viral vector vaccines; however, in these studies, viral vector vaccines accounted for a very small proportion, and they did not conduct further analysis on the types of vaccines [55, 12]. Data from Korea showed that pregnant women administered viral vector vaccines had higher rates of miscarriage and other obstetric complications than those administered mRNA vaccines. As indicated by the authors, this may be related to the older age of the viral vector vaccine group. Due to the inability to obtain detailed age information, the authors were unable to adjust for the impact of age [4]. The main vaccine type in India is the adenovirus vaccine, and a prospective report on adverse events in pregnant women vaccinated with the adenovirus has been published. Unfortunately, these studies focused only on adverse events that were not directly related to pregnancy [29]. We look forward to further research on inactivated viruses, viral vectors, recombinant protein vaccines, and other types of vaccines to supplement the safety evidence for COVID-19 vaccine during pregnancy.

It should be noted that that most investigators did not indicate the conception modes of these women, only Cao et al. [15], and Ma et al. [52], showed it, which were pregnant after IVF treatment and natural conception, respectively. Previous studies have reported that compared with natural conception, pregnancy after IVF treatment may be associated with a higher rate of abnormal pregnancy outcomes [82]. Therefore, if these studies can distinguish the conception patterns of these pregnant women, the results will have more practical significance. In addition, many of the above studies have reported the proportion of COVID-19 infection during pregnancy both in vaccinated and unvaccinated group, but they did not further analyze whether this infection affected their conclusions. In fact, it had been reported in the literature that the proportion of adverse pregnancy outcomes of pregnant women infected with COVID-19 had increased [25].

## 6. Conclusions

We indirectly determined the effect of the COVID-19 vaccine on male fertility based on its effects on male semen parameters. Although a few studies have suggested that the COVID-19 vaccine may cause a certain degree of fluctuation within the range of physiological changes, it has not yet reached the level of affecting the partner’s pregnancy probability. It is believed that receiving the COVID-19 vaccine does not affect fertility in non-pregnant women. Further research is needed to determine whether there are potential short-term adverse effects of vaccination. Vaccination during any trimester is considered safe in pregnant women.

## Data Availability

The datasets generated and/or analyzed during the current study are available in the MEDLINE repository. https://pubmed.ncbi.nlm.nih.gov/
